# T Cell Activation Triggers Calcium-Dependent Expression of the Aging Marker p16INK4a

**DOI:** 10.1093/geroni/igaf122.4016

**Published:** 2025-12-31

**Authors:** Noah Lepola, Xiangnan Guan, Christin Burd

**Affiliations:** Ohio State University, Columbus, Ohio, United States; Ohio State University, Columbus, Ohio, United States; Ohio State University, Columbus, Ohio, United States

## Abstract

p16INK4a is a senescence-associated cell cycle inhibitor and aging biomarker. Gerontogenic factors, including chemotherapy, smoking, and physical inactivity lead to durable increases in human peripheral blood T cell (PBTL) p16INK4a levels. However, the mechanisms that regulate p16INK4a in T cells remain uncertain. Here, we show that T cell activation signals induce p16INK4a mRNA and protein in mouse and human T cells. This induction is primarily driven by CD3 signaling, and is further enhanced by IL-2, but not by CD28 co-stimulation. Using luciferase reporter assays in Jurkat cells, we identify a predicted NFAT/Sp1 binding site in the p16INK4a promoter that is required for reporter activation following T cell stimulation. Furthermore, cyclosporine A-mediated inhibition of calcium signaling and NFAT activation limits endogenous p16INK4a expression during T cell stimulation. These findings suggest that p16INK4a is primarily regulated by calcium-dependent NFAT signaling downstream of CD3 engagement in T cells.

